# Mobilization of Endothelial Progenitors by Recurrent Bacteremias with a Periodontal Pathogen

**DOI:** 10.1371/journal.pone.0054860

**Published:** 2013-01-23

**Authors:** Moritz Kebschull, Manuela Haupt, Søren Jepsen, James Deschner, Georg Nickenig, Nikos Werner

**Affiliations:** 1 Department of Periodontology, Operative and Preventive Dentistry, University of Bonn, Bonn, Germany; 2 Department of Internal Medicine II, University of Bonn, Bonn, Germany; University of Padova, Medical School, Italy

## Abstract

**Background:**

Periodontal infections are independent risk factors for atherosclerosis. However, the exact mechanisms underlying this link are yet unclear. Here, we evaluate the in vivo effects of bacteremia with a periodontal pathogen on endothelial progenitors, bone marrow-derived cells capable of endothelial regeneration, and delineate the critical pathways for these effects.

**Methods:**

12-week old C57bl6 wildtype or toll-like receptor (TLR)-2 deficient mice were repeatedly intravenously challenged with 10^9^ live *P. gingivalis* 381 or vehicle. Numbers of Sca1+/flk1+ progenitors, circulating angiogenic cells, CFU-Hill, and late-outgrowth EPC were measured by FACS/culture. Endothelial function was assessed using isolated organ baths, reendothelization was measured in a carotid injury model. RANKL/osteoprotegerin levels were assessed by ELISA/qPCR.

**Results:**

In wildtype mice challenged with intravenous *P.gingivalis*, numbers of Sca1+/flk1+ progenitors, CAC, CFU-Hill, and late-outgrowth EPC were strongly increased in peripheral circulation and spleen, whereas Sca1+/flk1+ progenitor numbers in bone marrow decreased. Circulating EPCs were functional, as indicated by improved endothelial function and improved reendothelization in infected mice. The osteoprotegerin/RANKL ratio was increased after *P. gingivalis* challenge in the bone marrow niche of wildtype mice and late-outgrowth EPC *in vitro*. Conversely, in mice deficient in TLR2, no increase in progenitor mobilization or osteoprotegerin/RANKL ratio was detected.

**Conclusion:**

Recurrent transient bacteremias, a feature of periodontitis, increase peripheral EPC counts and decrease EPC pools in the bone marrow, thereby possibly reducing overall endothelial regeneration capacity, conceivably explaining pro-atherogenic properties of periodontal infections. These effects are seemingly mediated by toll-like receptor (TLR)-2.

## Introduction

Cardiovascular diseases are the leading cause of mortality in the western world. Their underlying pathological condition is atherosclerosis [1]. Risk factors for the development or acceleration of atherosclerosis include established predictors identified in the Framingham study [2], but also chronic infections, most notably periodontitis, a highly prevalent chronic inflammatory condition of the tooth-supporting tissues caused by specific periodontal pathogens in a susceptible host [3], [4].

There is ample evidence from epidemiological studies suggesting that periodontal infections are an independent risk factor for atherosclerosis [5]–[7]. Despite the overall modest association, the consistency of data across different study populations, exposures and outcome variables suggests that these findings are not spurious or attributable to confounders.

In the past, several potential mechanisms for a periodontal-cardiovascular link have been proposed (for review, see [6], [8]). These are conceptually based on the fact that the sizable ulcerated epithelium of the periodontal pockets [9] mediates persistent, recurrent bacteremia with pathogens (for review, see [10]). The mechanisms include direct effects of periodontal pathogens or their components on vascular cells, auto-immune reactions, and oxidative stress. A causative link of infections with activation of the innate immune system, and increased atherogenesis is strongly suggested by recent studies demonstrating the necessity of pattern-recognizing receptors, e.g. the Toll-like receptors, for atherosclerotic lesion formation [11]–[16]. Specifically, it was shown that TLR2, the receptor recognizing a principal pathogen in human periodontitis, the gram-negative anaerobe *Porphyromonas gingivalis,* is critical for both the effects of the pathogen in the oral cavity [17], [18] and in atherogenesis [19], [20]. Interaction of *P. gingivalis* fimbriae with TLR2 is necessary to mediate invasion of the pathogen into endothelial cells, where it was shown to persist and replicate [21], and eventually induce endothelial dysfunction [22].

A critical first step in atherogenesis is the activation of vascular endothelial cells and the development of endothelial dysfunction with subsequent apoptosis of endothelial cells [23]. Cross-sectional studies have demonstrated increased endothelial dysfunction in otherwise healthy patients with periodontitis [24], [25]. Periodontal therapy could improve endothelial dysfunction [26]. However, the pathways underlying these effects are not yet fully understood.

Vascular health is maintained by healthy endothelium that can in part be regenerated by circulating endothelial-regenerating cells (e.g. Sca1+/flk1+ progenitors) [27]. Numbers of these regenerating cells are associated with endothelial function [28] and cardiovascular outcomes [29]. Impaired endothelial regeneration after endothelial cell damage – as known to be elicited by periodontal pathogens [30] - is closely connected to the development of atherosclerotic lesions [31].

However, no mechanistic studies evaluating the effect of periodontal infections on endothelial regeneration have been conducted so far. The data available to date are limited to a single, cross-sectional study showing increased endothelial progenitor cell (EPC) counts in otherwise healthy periodontal patients when compared to controls without periodontitis [32], and an intervention study in the same population that showed a decrease of CD34 positive cells by periodontal therapy [33]. Importantly, these studies were neither designed nor suitable to prove causality, or to investigate the underlying mechanisms of a potential association.

Therefore, in this study we evaluated the effects of infection with the periodontal model pathogen *P. gingivalis* on numbers of different endothelial progenitor cell populations in an *in vivo* model. We aimed to evaluate whether progenitor cell numbers were in fact higher in infected groups than controls, and to determine the biological significance of this finding.

## Materials and Methods

### Ethics Statement

All animal experiments were performed in accordance with institutional guidelines and the German animal protection law. The study protocol was approved by the appropriate authority (North-Rhine Westphalia State Environment Agency (*Landesamt für Natur, Umwelt und Verbraucherschutz*/LANUV), Recklinghausen,Germany, permit no #8.87-50.10.35.08.013).

### Mice

Female, 12-week-old C57bl6 mice (Charles River, Sulzfeld, Germany) or age- and gender-matched TLR2−/− mice (a kind gift of Dr. Sabine Specht, Bonn) were used for this study. The animals were maintained in a 22°C room with a 12-hour light/dark cycle and received chow and water *ad libitum*. The mice were killed at day 12, and blood and tissue samples were recovered immediately. To account for potential gender-specific effects in the wildtype mice, we also tested several age-matched male mice, with very similar results than in female mice (data not shown).

### Bacteria

*Porphyromonas gingivalis* strain 381 (a kind gift of Dr. Evie Lalla, New York, NY, USA) was cultured under anaerobic conditions, as described previously [34].

### *In vivo* Infection (Bacteremia Model)

2×10^9^ live *P. gingivalis* resuspended in 200 µl saline or saline alone (control) were injected into the tail veins on days 0, 2, 4, 6, 8, and 10.

### Flow Cytometry

Peripheral blood was collected from the inferior vena cava at sacrifice. Bone marrow cells were flushed from both femurs using sterile saline.

After red blood cell lysis (BD Pharm Lyse, BD, Heidelberg, Germany) and blocking of the Fcγ II/III receptors (CD16/CD32, BD), the viable lymphocyte population was assessed for the expression of Sca-1-FITC (BD Pharmingen) and vascular endothelial growth factor receptor-2 coupled to PE (VEGFR2/flk-1, BD). Isotype-identical antibodies served as controls (BD). Analyses were run on a BD FACScalibur flow cytometer (BD), data were analyzed using FloJo (Treestar, Ashland, OR, USA).

Data were presented as %gated, relative to levels found in control mice.

### Preparation of Spleen-derived Mononuclear Cells

Spleens were minced and gently homogenized. The resulting single cell suspension was fractionated using Ficoll (Percoll, Biochrom, Berlin, Germany) gradient centrifugation.

### Preparation of Circulating Angiogenic Cells (CAC)

1×10^6^ spleen-derived mononuclear cells were seeded into fibronectin-coated (Sigma-Aldrich, St. Louis, MO, USA) 24-well plates in 500 µl of endothelial basal medium 2 (Lonza) with supplements. After 7 days in culture, cells were assayed for Dil-Ac-LDL uptake and lectin staining (UEA-1, Sigma-Aldrich). Per well, 5 high-power fields were analyzed by a blinded observer (author MH) for Dil-Ac-LDL+/lectin+ staining.

### Preparation of CFU–Hill

CFU-Hill were cultured from splenic MNCs, as described [35], [36]. In brief, 1×10^7^ cells were seeded on 6 cm dishes in complete EBM-2 medium, after 48 hours, 1×10^6^ non-adherent cells were collected and replated on fibronectin-coated 24-well plates for 7 days. CFU-Hill were counted by a blinded observer (author MH) using a phase-contrast microscope using a mosaic of 10×10 high power fields.

### Preparation of Late-outgrowth EPC

Late-outgrowth EPC were grown from splenic MNCs, as described [37], [38]. In brief, 1×10^7^ cells were seeded in complete EBM-2 medium on a 6 cm dish, non-adherent cells were removed after 48 hours, and cells were cultured for a total of 21 days. Colonies were identified by visual inspection using phase-contrast microscopy. Results of the dichotomous decision (presence of differentiated colonies) by a blinded observer (author MH) were statistically tested using Fisheŕs exact test.

### *In vitro* Infection

1×10^5^ phenotyped [39] late-outgrowth EPC were seeded in 6 cm dishes in EBM-2 medium with growth supplements, but without antibiotics. 5×10^6^, 1×10^7^, or 5×10^7^ live *P. gingivalis*381 were added, corresponding to a multiplicity-of-infection (MOI) of 50, 100, or 500 bacteria per EPC. The infection was maintained for 24 hours.

### ELISA

Bone marrow supernatants were produced by flushing both femurs with chilled saline and subsequent removal of bone marrow cells by centrifugation. Levels of RANKL and osteoprotegerin protein were assessed using commercially available ELISAs (#DY462 and #DY805, R&D Systems, Abingdon, UK) and normalized for total protein content, as assessed by Bradford assay.

### Quantitative RT-PCR

Total RNA was isolated using Trizol reagent (Invitrogen, Carlsbad, CA) and subsequent spin-column purification (RNeasy Mini Kit, Qiagen, Hilden, Germany), reverse-transcribed using Superscript III (Invitrogen) and analyzed using Taqman chemistry (Applied Biosystems, Foster City, CA, USA) using the probes Mm00441906_m1 for RANKL, Mm01205928_m1 for osteoprotegerin, and Mm99999915_g1 for GAPDH on a ABi 7500 Fast cycler (Applied Biosystems). Data were analyzed using the ΔΔct method.

### Aortic Ring Preparation and ex vivo Measurement of Endothelial Function

To assess endothelial function ex vivo, vasoconstriction and endothelium-dependent and –independent vasodilation were measured in isolated organ baths, as described previously [40]. In brief, the thoracic aorta was carefully dissected, adventitial tissue was removed, and 3 mm segments were prepared for investigation (3–4 replicates/mouse) in organ baths filled with oxygenated modified Tyrode buffer at 37°C. After administration of a resting tension of 10 mN, drugs were added in increasing concentrations and cumulative concentration response curves were recorded. We used 20 and 40 mM KCl and 1 nM –10 µM phenylephrine to induce aortic ring contraction. 10 nM –100 µM carbachol were then added to assess endothelium-dependent vasodilation after precontraction with phenylephrine. Finally, endothelium-independent vasodilation was assessed by application of 1 nM –10 µM nitroglycerine. At each drug concentration, a plateau phase was observed before addition of a higher dose of the drug. Before addition of the next substance, drugs were washed out.

### Reendothelization after Defined Carotid Artery Injury

Reendothelization was assessed in an electric injury model of the common carotid artery, as described previously [41]. In brief, on day 7 of the experimental protocol, a defined area of 4 mm length of the distal common carotid artery was denuded of endothelium using a bipolar microregulator.

The extent of endothelial repair was measured after 5 days by staining of the denuded areas by intravenous injection of 50 µl Evan’s blue dye (5% in saline). The yet unrepaired area – stained blue by the dye – was quantified by a blinded observer (author MH) using a calibrated stereomicroscope and expressed as percentage of the total injured area. Data are presented relative to the mean unrepaired are in sham-infected control animals.

### Statistical Analysis

All data were analyzed using GraphPad Prism V (GraphPad, San Diego, CA, USA). Normality of data was assessed using d’Agostino K^2^ tests, where appropriate. For normally distributed data, in comparisons of two groups, two-tailed, unpaired or paired (analysis of circulating angiogenic cells) t-tests were used, for comparisons of three or more groups, one-way ANOVA and *post-hoc* Tukey tests or two-way ANOVA with *post-hoc* Bonferroni tests were utilized. Late-outgrowth EPC experiments yielding dichotomous decisions were analyzed by Fisheŕs exact test. All data are presented as means ± SEM. A p-value of <0.05 was considered significant.

## Results

**Figure 1:**
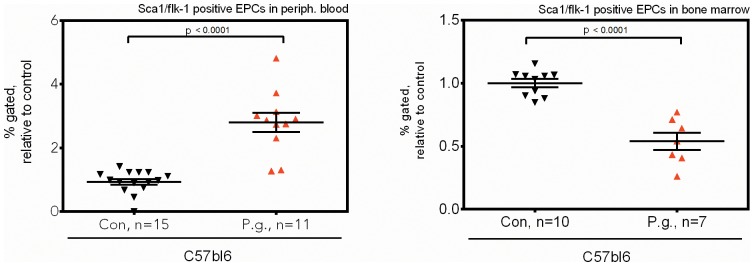
Bacteremia with P. gingivalis leads to mobilization of Sca1/flk1 progenitors from the bone marrow into peripheral blood of wildtype mice. Sca-1/flk1 progenitor cells (EPC) were quantified by flow cytometry in peripheral blood (left panel) or bone marrow (right panel) of C56bl6 wildtype mice after intravenous application of the periodontal pathogen *P. gingivalis* or saline (control). Data are presented as mean percentages of gated cells ± SEM normalized to control, statistical testing was performed using unpaired t-tests.

In C57bl6 wildtype animals, recurrent bacteremia with *P. gingivalis* did not impair clinical status of the animals, but lead to strongly increased peripheral count of Sca1+/flk1+ progenitors (control vs. test: 1.0±0.09 vs. 2.80±0.30, p<0.0001) with concomitantly decreased counts in bone marrow (control vs. test: 1.0±0.03 vs. 0.54±0.06, p<0.0001; figure 1). This increase in progenitor counts was dependent on the numbers of periodontal pathogens injected (data not shown).

**Figure 2:**
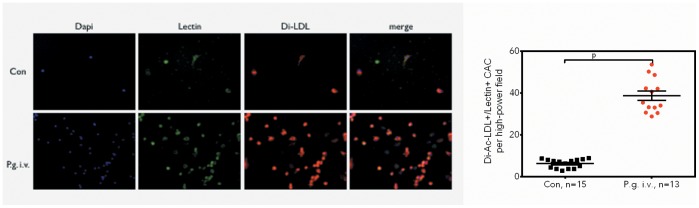
Bacteremia with P. gingivalis leads to increased numbers of circulating angiogenic cells (CACs) in wildtype mice. Strongly increased counts of Dil-Ac-LDL+/Lectin+ spleen-derived CACs in *P. gingivalis* infected mice. Data are presented as means of five high-power fields/mouse ± SEM, statistical testing was performed using paired t-tests to account for day-to-day staining variability.

**Figure 3:**
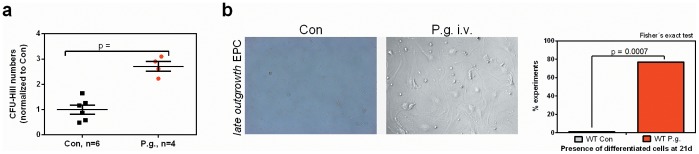
Bacteremia with P. gingivalis leads to increased numbers of CFU-Hill and late-outgrowth EPC in wildtype mice. (a) Increased numbers of CFU-Hill, (b) high proportion of presence of differentiated late-outgrowth EPC in *P. gingivalis* infected mice. Data are presented as mean colony counts ± SEM relative to controls (CFU-Hill) or as numbers of experiments yielding differentiated colonies (late EPC). Data were analyzed using unpaired t-tests (CFU-Hill) or Fisheŕs exact test (late EPC).

Spleen-derived early and late-outgrowth endothelial progenitor population counts were also increased by *P. gingivalis* bacteremia. Specifically, numbers of Dil-Ac-LDL+/lectin+ circulating angiogenic cells (CAC) were increased more than 5-fold in the infected group (control vs. test: 6.2±0.55 vs. 38.66±2.28 double-positive cells/HPF, p<0.0001; figure 2), numbers of CFU-Hill were increased (control vs. test: 1.00±0.18 vs. 2.71±0.19 CFU/HPF (normalized to control), p = 0.0002; figure 3a), and almost all infected mice yielded differentiated late-outgrowth EPC, whilst those in the control group showed no late-outgrowth EPC development after 21 days in culture (figure 3b).

To assess the biological functional relevance of the observed strong increases in counts of the different endothelial progenitor populations, we determined the impact of a recurrent bacteremia and subsequent progenitor mobilization on endothelial function and reendothelization.

**Figure 4:**
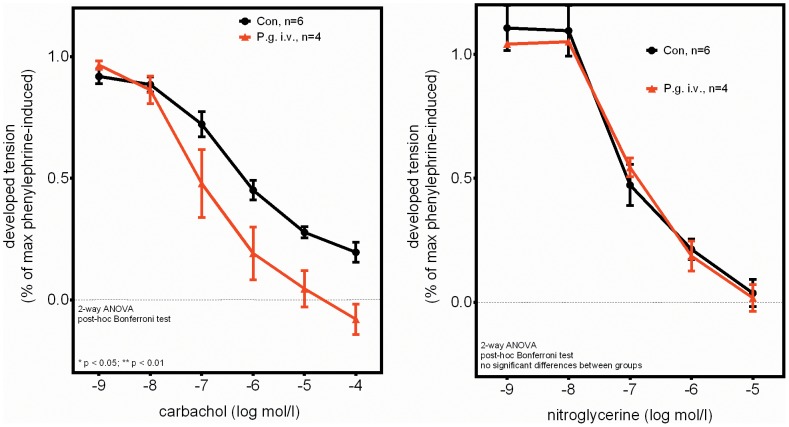
Improved endothelium-dependent vasodilation in P. gingivalis infected mice (left panel). No difference between groups in endothelium-independent vasodilation (right panel). Data are presented as means of 3–4 aortic ring preparations/mouse ± SEM. Statistical testing was performed using 2-way ANOVA and post-hoc Bonferroni tests.

Endothelium-dependent vasodilation, a measure of endothelial function, was significantly improved in the bacteremia group (figure 4, left panel). Endothelium-independent vasodilation on the other hand was similar in the bacteremia and the control group (figure 4, right panel).

**Figure 5:**
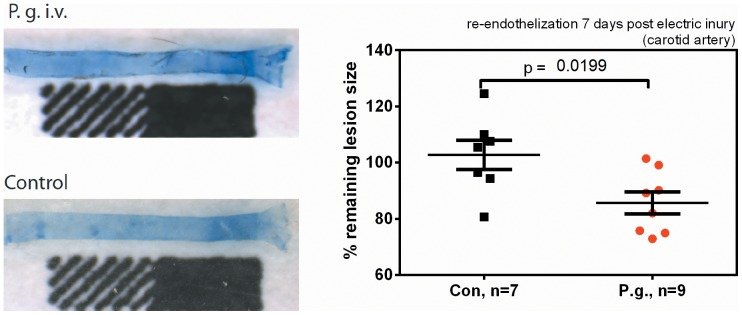
Improved reendothelization in P. gingivalis infected mice 5 days after electric injury of the common carotid artery. Data are expressed as proportions of non-regenerated to regenerated areas relative to saline-treated controls. Micrographs show representative results, dark blue areas mark denuded areas. Data are presented as means ± SEM, statistical testing was performed using an unpaired t-test.

Similarly, reendothelization after electric/thermic denudation of the endothelium of the common carotid artery was improved in the bacteremia group characterized by high numbers of peripheral progenitors (figure 5).

Lastly, we assessed how critical mediators of bone marrow cell mobilization were affected by *P. gingivalis* infection and the subsequent strong mobilization of progenitors from bone marrow into peripheral blood.

**Figure 6:**
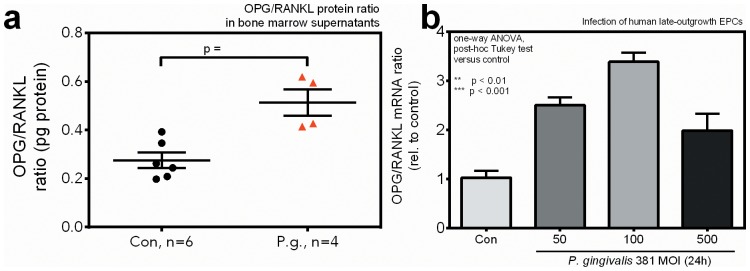
Increased osteoprotegerin/RANKL protein ratio within the bone marrow nice in P. gingivalis infected mice (a), increased osteoprotegerin/RANKL mRNA ratio in P. gingivalis infected late-outgrowth EPC *in vitro* (b). Osteoprotegerin induces bone marrow cell retention and expansion, RANKL triggers mobilization. Increased osteoprotegerin/RANKL ratios may counter act a strong mobilization and depletion of bone marrow EPC. Data are presented as means ± SEM, statistical testing was performed using an unpaired t-test (protein data) and ANOVA and post-hoc Tukey test (mRNA data). MOI, multiplicity-of-infection (ratio of prokaryotic/eukaryotic cells).

In the bone marrow niche, we found an increased osteoprotegerin/RANKL protein ratio in *P. gingivalis* infected mice (control vs. test: 0.27±0.03 vs. 0.51±0.05, p = 0.0035; figure 6a). In line with this observation, an increased osteoprotegerin/RANKL mRNA ratio (+339±0.18% at MOI 100, p<0.0001) was observed in *P. gingivalis* infected late-outgrowth EPC *in vitro* (figure 6b).

**Figure 7:**
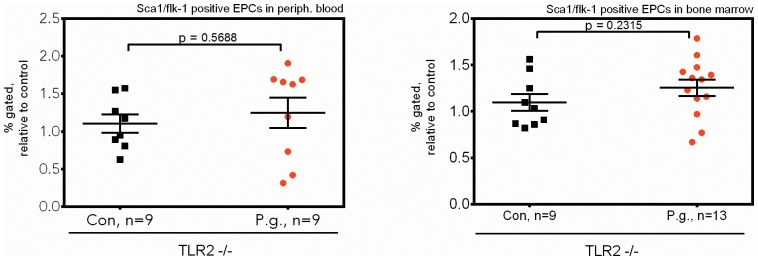
Sca1+/flk1+ progenitor mobilization is TLR2-dependent. In mice deficient in toll-like receptor (TLR)-2, the receptor primarily mediating the invasion of *P. gingivalis* into host cells, no increased mobilization of Sca1+/flk1+ progenitors was observed. Data are presented as mean percentages of gated cells ± SEM normalized to control, statistical testing was performed using unpaired t-tests.

**Figure 8:**
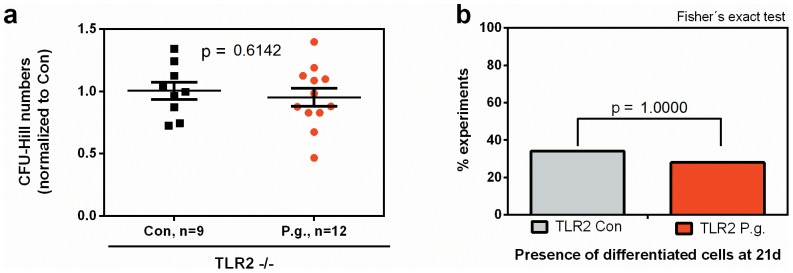
CFU-Hill and late-outgrowth mobilization is TLR2-dependent. No significant differences in CFU-Hill numbers (a) and the proportion of presence of differentiated late-outgrowth EPC (b) in *P. gingivalis* infected TLR2-deficient mice. Data are presented as mean colony counts ± SEM relative to controls (CFU-Hill) or as numbers of experiments yielding differentiated colonies (late EPC). Data were analyzed using unpaired t-tests (CFU-Hill) or Fisheŕs exact test (late EPC).

**Figure 9:**
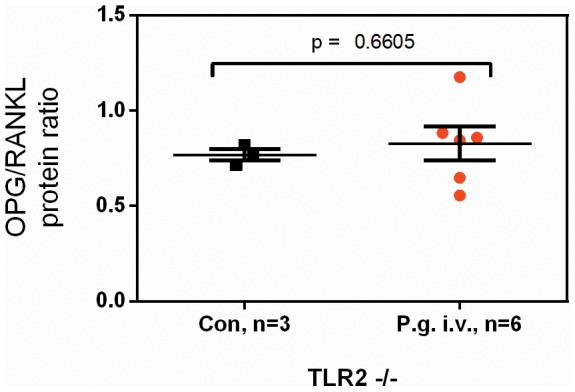
No differences in osteoprotegerin/RANKL ratio in the bone marrow niche of P. gingivalis infected vs. control mice deficient in TLR2. Data are given as means ± SEM, data were analyzed using an unpaired t-test.

Finally, we evaluated the molecular pathway underlying the demonstrated mobilization and concomitant depletion of functional endothelial progenitors by recurrent bacteremia with the periodontal pathogen *P. gingivalis*. Unlike most gram-negative species, *P. gingivalis* was described to primarily utilize toll-like receptor (TLR)-2, rather than TLR4, to invade into host cells and exert its primary biological effects. Indeed, in mice deficient in TLR2, we could not observe the pronounced mobilization found in C56bl6 wildtype mice (figures 7&8). In line with these observations, the aforementioned increase in osteoprotegerin/RANKL protein ratios in the bone marrow niche of *P. gingivalis* infected mice was not found in the absence of TLR2 (figure 9). These data indicate that the observed biological effects on progenitor mobilization are in fact primarily mediated by interactions of *P. gingivalis* with the TLR2 receptor.

## Discussion

Here, we show that recurrent bacteremias with the periodontal model pathogen *P. gingivalis* induce Sca1+/flk1+ endothelial progenitor mobilization from the bone marrow into the peripheral circulation *in vivo* and result in higher levels of both early and late EPC, distinct progenitor cell subtypes with dissimilar properties [42]. These data corroborate reports from a cross-sectional study showing higher peripheral EPC counts in otherwise healthy periodontal patients [32]. Increased levels of endothelial progenitors have also been associated with other inflammatory conditions exhibiting similarities in pathobiological mechanisms with periodontal infections, such as rheumatoid arthritis [43], or in situations of acute tissue damage, such as myocardial infarction [44]–[47], percutaneous coronary intervention [48], or excessive exercise [49].

The infection-induced mobilization was dependent on the presence of toll-like receptor 2 (TLR2), the receptor primarily mediating the invasion of *P. gingivalis* into host cells [11], seemingly a prerequisite for exerting its biological effects. These data are in line with prior observations of reduced periodontal infection-mediated atherosclerosis in a mouse model deficient in TLR2 [19], [20] and point to a potential role for TLR2 as a therapeutic target in host modulation [50]. Still, it needs to be noted that since TLRs play a vital role in host defense, there exist several challenges that future TLR-targeting drugs need to overcome [51]. Importantly, our data suggesting a specific reaction towards the periodontal pathogen also corroborate reports showing that unspecific acute systemic inflammation *per se* does not lead to increased mobilization of endothelial progenitors [52].

The beneficial effects of acute bacteremia with the periodontal pathogen on endothelial function and reendothelization point to a functional relevance of mobilized endothelial progenitors. Our group has previously demonstrated that increased levels of endothelial progenitors by systemic transfusion in fact significantly improve endothelial function [40].

In addition to the hitherto demonstrated beneficial effect of regenerating progenitors, the observed significantly improved endothelial-dependent vasodilation in infected mice could also be attributed in part to the excessive vasodilatation in sepsis [53]. However, it must be noted (i) that our treatment regimen did not at all induce a septic shock, a prerequisite for abundant NO production and vasodilatory state, and (ii) that we also demonstrated an improved re-endothelization in the infected group, an outcome independent of endothelial vasodilatory state.

Thus, the observed enhanced mobilization of endothelial progenitors leads to a short-term functional improvement, likely acting as a counter-measure against pathogen-mediated endothelial damage. However, since during the course of periodontal infections, bacteremia with periodontal pathogens is a frequent event [10], recurrent mobilization of bone marrow-derived cells may conceivably deplete bone marrow pools over time, thereby reducing the overall regenerative potential and facilitating atherogenesis. Still, to adequately test for the long-term consequences of our observations in the present, acute bacteremia model, appropriate atherosclerosis models need to be employed [54].

To further assess the mobilization of progenitors, we examined in mice infected with *P. gingivalis* the levels of RANKL, a cytokine triggering mobilization [55] and osteoprotegerin, a decoy receptor for RANKL that is known to mediate bone marrow cell expansion and retention that was recently established as a biomarker for cardiovascular prognosis and mortality [56], [57]. Importantly, we found increased osteoprotegerin/RANKL ratios in the bone marrow niche of infected wildtype mice. In this work, we have defined this niche as a ‘cellular and molecular microenvironment that regulates (…) the engagement of specific programs in response to stress’, as proposed by Ehninger and Trumpp [58]. Contrastingly, in TLR2-deficient mice showing no mobilization, the ratio was unchanged. In this context, it needs to be mentioned that the number of control animals in this comparison was rather low (n = 3) due to the sparse availability of the knockout animals. This finding is unexpected, since an increased ratio is indicative for increased retention and decreased mobilization of bone marrow cells [55]. Here, is could possibly act as a counter measure against the conceivable bone marrow cell depletion by recurrent bacteremias. In fact, our group has previously shown that increased systemic progenitor levels in response to physical training are accompanied by similarly increased, and not diminished, levels in the bone marrow [59].

In line with these observations in vivo, we could demonstrate in vitro that infection of human late-outgrowth EPC with *P. gingivalis* also increases the osteoprotegerin/RANKL ratio. Interestingly, the increase was less pronounced at multiplicities of infection in the range associated with apoptotic changes in endothelial cells [30], an observation in line with reports of decreased osteoprotegerin production in endothelial cells with activated p53 [60]. Increased systemic osteoprotegerin/RANKL ratios after intravascular application of *P. gingivalis* LPS have been reported before [61].

Interestingly, this is in contrast to the well-established osteoclastic phenotype dominated by increased RANKL over osteoprotegerin levels [62] that is found in periodontal lesions that are primarily characterized by bone resorption [63]. We attribute this discrepancy of effects triggered by *P. gingivalis* to the apparent strong differences in cellular composition of the different tissue compartments. The pathogen in the systemic circulation is most likely to encounter endothelial cells and progenitors or vascular smooth muscle cells, potent producers of osteoprotegerin when activated [64], whilst in the periodontal tissues, the pathogens are in intimate contact with bone stromal cells and infiltrating mononuclear cells, such as T-cells, all of those are known to predominantly produce RANKL [65], [66].

On the other hand, osteoprotegerin, but not RANKL, was recently shown to induce endothelial cell and ECFC activation and to improve microvessel formation in vitro [67], [68] – therefore, it must also be considered that the observed increase of the systemic osteoprotegerin/RANKL ratio might as well constitute another counter-measure against vascular damage inflicted by the pathogen.

When judging the inferences drawn from our data, it must be, however, noted that the utilized acute model of bacteremia with a periodontal model pathogen is neither a model of atherosclerosis nor of periodontitis. It merely mimics the bacteremia known to be a feature of active periodontal disease [6], [10], [69], [70], and allows for exact determination of the effects exerted by these bacteria on bone marrow-derived progenitor mobilization.

An oral model of periodontal infection in atherosclerosis-prone mice [54] is more likely to adequately mimic human periodontal disease and its effects on atherosclerosis progression. However, these models all rely on genetically modified mice prone to develop atherosclerosis in due course, potentially not ideal models to shed light onto the underlying pathobiology of associations found in otherwise healthy subjects suffering from periodontitis.

Subsequent studies in adequate models of chronic challenge with periodontal pathogens are necessary to evaluate whether the observed strong mobilization of endothelial progenitors from the bone marrow into the peripheral circulation induced by bacteremia with *P. gingivalis* will in fact lead to depletion of progenitor pools and subsequently reduced overall regeneration capacity. Alternatively, it is possible that the acute shortage of progenitors in the bone marrow is effectively countered by increasing osteoprotegerin/RANKL levels leading to stem cell expansion and retention, eventually resulting in increased EPC levels in peripheral blood and bone marrow nice, as found after repeated physical exercise [59].

These studies will unequivocally show whether, to what extent, and by what mechanisms endothelium-regenerating cells are involved in increased atherogenesis mediated by periodontal infections.

Taken together, we show that in an acute model of periodontal infection recurrent bacteremias lead to strong, TLR2-dependent mobilization of endothelial progenitors from the bone marrow to the circulation. In the short term, these cells improve endothelial function and reendothelization. Long-term studies in atherosclerosis models are needed to determine whether this recurrent mobilization is relevant for the reported increased atherosclerosis in periodontitis.

## References

[1] Libby P, Ridker PM, Maseri A (2002) Inflammation and atherosclerosis. Circulation 105: 1135–1143. 11877368 10.1161/hc0902.104353

[2] Borden WB, Davidson MH (2009) Updating the assessment of cardiac risk: beyond Framingham. Rev Cardiovasc Med 10: 63–71. 19593318

[3] Darveau R (2010) Periodontitis: a polymicrobial disruption of host homeostasis. Nat Rev Microbiol 8: 481–490. 20514045 10.1038/nrmicro2337

[4] Pihlstrom BL, Michalowicz BS, Johnson NW (2005) Periodontal diseases. Lancet 366: 1809–1820. 16298220 10.1016/S0140-6736(05)67728-8

[5] Dietrich T, Jimenez M, Krall Kaye EA, Vokonas PS, Garcia RI (2008) Age-dependent associations between chronic periodontitis/edentulism and risk of coronary heart disease. Circulation 117: 1668–1674. 18362228 10.1161/CIRCULATIONAHA.107.711507 PMC2582144

[6] Kebschull M, Demmer RT, Papapanou PN (2010) “Gum bug, leave my heart alone!”–epidemiologic and mechanistic evidence linking periodontal infections and atherosclerosis. J Dent Res 89: 879–902. 20639510 10.1177/0022034510375281 PMC3318075

[7] Lockhart PB, Bolger AF, Papapanou PN, Osinbowale O, Trevisan M, et al. (2012) Periodontal Disease and Atherosclerotic Vascular Disease: Does the Evidence Support an Independent Association?: A Scientific Statement From the American Heart Association. Circulation. 10.1161/CIR.0b013e31825719f3 22514251

[8] Hayashi C, Gudino CV, Gibson FC 3rd, Genco CA (2010) Review: Pathogen-induced inflammation at sites distant from oral infection: bacterial persistence and induction of cell-specific innate immune inflammatory pathways. Mol Oral Microbiol 25: 305–316. 20883220 10.1111/j.2041-1014.2010.00582.x PMC2951292

[9] Hujoel PP, White BA, Garcia RI, Listgarten MA (2001) The dentogingival epithelial surface area revisited. J Periodontal Res 36: 48–55. 11246704 10.1034/j.1600-0765.2001.00011.x

[10] Iwai T (2009) Periodontal bacteremia and various vascular diseases. J Periodontal Res 44: 689–694. 19874452 10.1111/j.1600-0765.2008.01165.x

[11] Hajishengallis G, Wang M, Bagby GJ, Nelson S (2008) Importance of TLR2 in early innate immune response to acute pulmonary infection with Porphyromonas gingivalis in mice. J Immunol 181: 4141–4149. 18768871 10.4049/jimmunol.181.6.4141 PMC2625304

[12] Liu X, Ukai T, Yumoto H, Davey M, Goswami S, et al (2008) Toll-like receptor 2 plays a critical role in the progression of atherosclerosis that is independent of dietary lipids. Atherosclerosis 196: 146–154. 17466307 10.1016/j.atherosclerosis.2007.03.025 PMC2243224

[13] Madan M, Amar S (2008) Toll-like receptor-2 mediates diet and/or pathogen associated atherosclerosis: proteomic findings. PLoS One 3: e3204. 18787704 10.1371/journal.pone.0003204 PMC2527517

[14] den Dekker WK, Cheng C, Pasterkamp G, Duckers HJ (2010) Toll like receptor 4 in atherosclerosis and plaque destabilization. Atherosclerosis 209: 314–320. 19900676 10.1016/j.atherosclerosis.2009.09.075

[15] Michelsen KS, Wong MH, Shah PK, Zhang W, Yano J, et al (2004) Lack of Toll-like receptor 4 or myeloid differentiation factor 88 reduces atherosclerosis and alters plaque phenotype in mice deficient in apolipoprotein E. Proc Natl Acad Sci U S A. 101: 10679–10684. 10.1073/pnas.0403249101 PMC489994 15249654

[16] Zimmer S, Steinmetz M, Asdonk T, Motz I, Coch C, et al (2011) Activation of endothelial toll-like receptor 3 impairs endothelial function. Circ Res 108: 1358–1366. 21493895 10.1161/CIRCRESAHA.111.243246

[17] Zhang P, Liu J, Xu Q, Harber G, Feng X, et al (2011) TLR2-dependent modulation of osteoclastogenesis by Porphyromonas gingivalis through differential induction of NFATc1 and NF-kappaB. J Biol Chem 286: 24159–24169. 21566133 10.1074/jbc.M110.198085 PMC3129197

[18] Burns E, Eliyahu T, Uematsu S, Akira S, Nussbaum G (2010) TLR2-dependent inflammatory response to Porphyromonas gingivalis is MyD88 independent, whereas MyD88 is required to clear infection. J Immunol 184: 1455–1462. 20042569 10.4049/jimmunol.0900378

[19] Gibson FC 3rd, Genco CA (2007) Porphyromonas gingivalis mediated periodontal disease and atherosclerosis: disparate diseases with commonalities in pathogenesis through TLRs. Curr Pharm Des 13: 3665–3675. 18220804 10.2174/138161207783018554

[20] Hayashi C, Madrigal AG, Liu X, Ukai T, Goswami S, et al (2010) Pathogen-mediated inflammatory atherosclerosis is mediated in part via Toll-like receptor 2-induced inflammatory responses. J Innate Immun 2: 334–343. 20505314 10.1159/000314686 PMC2895755

[21] Rodrigues PH, Belanger M, Dunn W Jr, Progulske-Fox A (2008) Porphyromonas gingivalis and the autophagic pathway: an innate immune interaction? Front Biosci 13: 178–187. 17981536 10.2741/2668

[22] Honda T, Oda T, Yoshie H, Yamazaki K (2005) Effects of Porphyromonas gingivalis antigens and proinflammatory cytokines on human coronary artery endothelial cells. Oral Microbiol Immunol 20: 82–88. 15720567 10.1111/j.1399-302X.2004.00193.x

[23] Sima AV, Stancu CS, Simionescu M (2009) Vascular endothelium in atherosclerosis. Cell Tissue Res 335: 191–203. 18797930 10.1007/s00441-008-0678-5

[24] Amar S, Gokce N, Morgan S, Loukideli M, Van Dyke TE, et al (2003) Periodontal disease is associated with brachial artery endothelial dysfunction and systemic inflammation. Arterioscler Thromb Vasc Biol 23: 1245–1249. 12763762 10.1161/01.ATV.0000078603.90302.4A

[25] Mercanoglu F, Oflaz H, Oz O, Gokbuget AY, Genchellac H, et al (2004) Endothelial dysfunction in patients with chronic periodontitis and its improvement after initial periodontal therapy. J Periodontol 75: 1694–1700. 15732873 10.1902/jop.2004.75.12.1694

[26] Tonetti MS, D’Aiuto F, Nibali L, Donald A, Storry C, et al (2007) Treatment of periodontitis and endothelial function. N Engl J Med 356: 911–920. 17329698 10.1056/NEJMoa063186

[27] Kirton JP, Xu Q (2010) Endothelial precursors in vascular repair. Microvasc Res 79: 193–199. 20184904 10.1016/j.mvr.2010.02.009

[28] Werner N, Wassmann S, Ahlers P, Schiegl T, Kosiol S, et al (2007) Endothelial progenitor cells correlate with endothelial function in patients with coronary artery disease. Basic Res Cardiol 102: 565–571. 17932708 10.1007/s00395-007-0680-1

[29] Werner N, Kosiol S, Schiegl T, Ahlers P, Walenta K, et al (2005) Circulating endothelial progenitor cells and cardiovascular outcomes. N Engl J Med 353: 999–1007. 16148285 10.1056/NEJMoa043814

[30] Roth GA, Ankersmit HJ, Brown VB, Papapanou PN, Schmidt AM, et al (2007) Porphyromonas gingivalis infection and cell death in human aortic endothelial cells. FEMS Microbiol Lett 272: 106–113. 17459112 10.1111/j.1574-6968.2007.00736.x

[31] Werner N, Nickenig G (2006) Clinical and therapeutical implications of EPC biology in atherosclerosis. J Cell Mol Med 10: 318–332. 16796802 10.1111/j.1582-4934.2006.tb00402.x PMC3933124

[32] Li X, Tse HF, Yiu KH, Jia N, Chen H, et al (2009) Increased levels of circulating endothelial progenitor cells in subjects with moderate to severe chronic periodontitis. J Clin Periodontol 36: 933–939. 19799717 10.1111/j.1600-051X.2009.01481.x

[33] Li X, Tse HF, Yiu KH, Li LS, Jin L (2011) Effect of periodontal treatment on circulating CD34(+) cells and peripheral vascular endothelial function: a randomized controlled trial. J Clin Periodontol 38: 148–156. 21133981 10.1111/j.1600-051X.2010.01651.x

[34] Pollreisz A, Huang Y, Roth GA, Cheng B, Kebschull M, et al (2010) Enhanced monocyte migration and pro-inflammatory cytokine production by Porphyromonas gingivalis infection. J Periodontal Res 45: 239–245. 19778327 10.1111/j.1600-0765.2009.01225.x

[35] Ito H, Rovira, II, Bloom ML, Takeda K, Ferrans VJ, et al (1999) Endothelial progenitor cells as putative targets for angiostatin. Cancer Res 59: 5875–5877. 10606226

[36] Hill JM, Zalos G, Halcox JP, Schenke WH, Waclawiw MA, et al (2003) Circulating endothelial progenitor cells, vascular function, and cardiovascular risk. N Engl J Med 348: 593–600. 12584367 10.1056/NEJMoa022287

[37] Ingram DA, Mead LE, Tanaka H, Meade V, Fenoglio A, et al (2004) Identification of a novel hierarchy of endothelial progenitor cells using human peripheral and umbilical cord blood. Blood 104: 2752–2760. 15226175 10.1182/blood-2004-04-1396

[38] Yoder MC, Mead LE, Prater D, Krier TR, Mroueh KN, et al (2007) Redefining endothelial progenitor cells via clonal analysis and hematopoietic stem/progenitor cell principals. Blood 109: 1801–1809. 17053059 10.1182/blood-2006-08-043471 PMC1801067

[39] Steinmetz M, Nickenig G, Werner N (2010) Endothelial-regenerating cells: an expanding universe. Hypertension 55: 593–599. 20083733 10.1161/HYPERTENSIONAHA.109.134213

[40] Wassmann S, Werner N, Czech T, Nickenig G (2006) Improvement of endothelial function by systemic transfusion of vascular progenitor cells. Circ Res 99: e74–83. 16990568 10.1161/01.RES.0000246095.90247.d4

[41] Brouchet L, Krust A, Dupont S, Chambon P, Bayard F, et al (2001) Estradiol accelerates reendothelialization in mouse carotid artery through estrogen receptor-alpha but not estrogen receptor-beta. Circulation 103: 423–428. 11157695 10.1161/01.cir.103.3.423

[42] Medina RJ, O’Neill CL, Sweeney M, Guduric-Fuchs J, Gardiner TA, et al (2010) Molecular analysis of endothelial progenitor cell (EPC) subtypes reveals two distinct cell populations with different identities. BMC Med Genomics 3: 18. 20465783 10.1186/1755-8794-3-18 PMC2881111

[43] Jodon de Villeroche V, Avouac J, Ponceau A, Ruiz B, Kahan A, et al (2010) Enhanced late-outgrowth circulating endothelial progenitor cell levels in rheumatoid arthritis and correlation with disease activity. Arthritis Res Ther 12: R27. 20158894 10.1186/ar2934 PMC2875661

[44] Massa M, Rosti V, Ferrario M, Campanelli R, Ramajoli I, et al (2005) Increased circulating hematopoietic and endothelial progenitor cells in the early phase of acute myocardial infarction. Blood 105: 199–206. 15345590 10.1182/blood-2004-05-1831

[45] Leone AM, Rutella S, Bonanno G, Abbate A, Rebuzzi AG, et al (2005) Mobilization of bone marrow-derived stem cells after myocardial infarction and left ventricular function. Eur Heart J 26: 1196–1204. 15734770 10.1093/eurheartj/ehi164

[46] Wojakowski W, Tendera M, Michalowska A, Majka M, Kucia M, et al (2004) Mobilization of CD34/CXCR4+, CD34/CD117+, c-met+ stem cells, and mononuclear cells expressing early cardiac, muscle, and endothelial markers into peripheral blood in patients with acute myocardial infarction. Circulation 110: 3213–3220. 15533859 10.1161/01.CIR.0000147609.39780.02

[47] Brehm M, Ebner P, Picard F, Urbien R, Turan G, et al (2009) Enhanced mobilization of CD34(+) progenitor cells expressing cell adhesion molecules in patients with STEMI. Clin Res Cardiol 98: 477–486. 19479183 10.1007/s00392-009-0021-5

[48] Banerjee S, Brilakis E, Zhang S, Roesle M, Lindsey J, et al (2006) Endothelial progenitor cell mobilization after percutaneous coronary intervention. Atherosclerosis 189: 70–75. 16806234 10.1016/j.atherosclerosis.2006.04.026

[49] Goussetis E, Spiropoulos A, Tsironi M, Skenderi K, Margeli A, et al (2009) Spartathlon, a 246 kilometer foot race: effects of acute inflammation induced by prolonged exercise on circulating progenitor reparative cells. Blood Cells Mol Dis 42: 294–299. 19233694 10.1016/j.bcmd.2009.01.003

[50] Hajishengallis G (2009) Toll gates to periodontal host modulation and vaccine therapy. Periodontol 2000 51: 181–207. 19878475 10.1111/j.1600-0757.2009.00304.x PMC2775135

[51] Cole JE, Mitra AT, Monaco C (2010) Treating atherosclerosis: the potential of Toll-like receptors as therapeutic targets. Expert Rev Cardiovasc Ther 8: 1619–1635. 21090937 10.1586/erc.10.149

[52] Padfield GJ, Tura O, Haeck ML, Short A, Freyer E, et al (2010) Circulating endothelial progenitor cells are not affected by acute systemic inflammation. Am J Physiol Heart Circ Physiol 298: H2054–2061. 20382859 10.1152/ajpheart.00921.2009 PMC2886634

[53] Sommers MS (2003) The cellular basis of septic shock. Crit Care Nurs Clin North Am 15: 13–25. 12597036 10.1016/s0899-5885(02)00046-1

[54] Lalla E, Lamster IB, Hofmann MA, Bucciarelli L, Jerud AP, et al (2003) Oral infection with a periodontal pathogen accelerates early atherosclerosis in apolipoprotein E-null mice. Arterioscler Thromb Vasc Biol 23: 1405–1411. 12816879 10.1161/01.ATV.0000082462.26258.FE

[55] Calvi LM, Adams GB, Weibrecht KW, Weber JM, Olson DP, et al (2003) Osteoblastic cells regulate the haematopoietic stem cell niche. Nature 425: 841–846. 14574413 10.1038/nature02040

[56] Venuraju SM, Yerramasu A, Corder R, Lahiri A (2010) Osteoprotegerin as a predictor of coronary artery disease and cardiovascular mortality and morbidity. J Am Coll Cardiol 55: 2049–2061. 20447527 10.1016/j.jacc.2010.03.013

[57] D’Amelio P, Isaia G, Isaia GC (2009) The osteoprotegerin/RANK/RANKL system: a bone key to vascular disease. J Endocrinol Invest 32: 6–9. 19724159

[58] Ehninger A, Trumpp A (2011) The bone marrow stem cell niche grows up: mesenchymal stem cells and macrophages move in. The Journal of Experimental Medicine 208: 421–428. 21402747 10.1084/jem.20110132 PMC3058583

[59] Laufs U, Werner N, Link A, Endres M, Wassmann S, et al (2004) Physical training increases endothelial progenitor cells, inhibits neointima formation, and enhances angiogenesis. Circulation 109: 220–226. 14691039 10.1161/01.CIR.0000109141.48980.37

[60] Secchiero P, Corallini F, Rimondi E, Chiaruttini C, di Iasio MG, et al (2008) Activation of the p53 pathway down-regulates the osteoprotegerin expression and release by vascular endothelial cells. Blood 111: 1287–1294. 18000166 10.1182/blood-2007-05-092031

[61] Lu HK, Yeh KC, Wu MF, Li CL, Tseng CC (2008) An acute injection of Porphyromonas gingivalis lipopolysaccharide modulates the OPG/RANKL system and interleukin-6 in an ovariectomized mouse model. Oral Microbiol Immunol 23: 220–225. 18402608 10.1111/j.1399-302X.2007.00415.x

[62] Graves D (2008) Cytokines that promote periodontal tissue destruction. J Periodontol 79: 1585–1591. 18673014 10.1902/jop.2008.080183

[63] Taubman MA, Valverde P, Han X, Kawai T (2005) Immune response: the key to bone resorption in periodontal disease. J Periodontol 76: 2033–2041. 10.1902/jop.2005.76.11-S.2033 16277573

[64] Zhang J, Fu M, Myles D, Zhu X, Du J, et al (2002) PDGF induces osteoprotegerin expression in vascular smooth muscle cells by multiple signal pathways. FEBS Lett 521: 180–184. 12067713 10.1016/s0014-5793(02)02872-7

[65] Belibasakis GN, Reddi D, Bostanci N (2010) Porphyromonas gingivalis Induces RANKL in T-cells. Inflammation. 10.1007/s10753-010-9216-1 20446027

[66] Reddi D, Bostanci N, Hashim A, Aduse-Opoku J, Curtis MA, et al (2008) Porphyromonas gingivalis regulates the RANKL-OPG system in bone marrow stromal cells. Microbes Infect 10: 1459–1468. 18789397 10.1016/j.micinf.2008.08.007

[67] Benslimane-Ahmim Z, Heymann D, Dizier B, Lokajczyk A, Brion R, et al. (2011) Osteoprotegerin, a new actor in vasculogenesis, stimulates endothelial colony-forming cells properties. J Thromb Haemost. 10.1111/j.1538-7836.2011.04207.x 21255246

[68] McGonigle JS, Giachelli CM, Scatena M (2009) Osteoprotegerin and RANKL differentially regulate angiogenesis and endothelial cell function. Angiogenesis 12: 35–46. 19105036 10.1007/s10456-008-9127-z PMC12490743

[69] Kinane DF, Riggio MP, Walker KF, MacKenzie D, Shearer B (2005) Bacteraemia following periodontal procedures. J Clin Periodontol 32: 708–713. 15966875 10.1111/j.1600-051X.2005.00741.x

[70] Lockhart PB, Brennan MT, Sasser HC, Fox PC, Paster BJ, et al (2008) Bacteremia associated with toothbrushing and dental extraction. Circulation 117: 3118–3125. 18541739 10.1161/CIRCULATIONAHA.107.758524 PMC2746717

